# Coexistence of Ankylosing Spondylitis and Neurofibromatosis Type 1

**DOI:** 10.1155/2016/4039801

**Published:** 2016-08-14

**Authors:** Baris Gundogdu, Servet Yolbas, Ahmet Yildirim, Murat Gonen, Suleyman Serdar Koca

**Affiliations:** ^1^Department of Rheumatology, Faculty of Medicine, Firat University, 23200 Elazig, Turkey; ^2^Department of Neurology, Faculty of Medicine, Firat University, 23200 Elazig, Turkey

## Abstract

Ankylosing spondylitis (AS) is a systemic disease primarily characterized by the inflammation of sacroiliac joints and axial skeleton. Neurofibromatosis type 1 (NF1) is a multisystem genetic disease which is characterized by cutaneous findings, most importantly café-au-lait spots and axillary freckling, by skeletal dysplasia, and by the growth of both benign and malignant nervous system neoplasms, most notably benign neurofibromas. In this case report, we present a 43-year-old male with AS and NF1.

## 1. Introduction

Ankylosing spondylitis (AS) is a chronic inflammatory disease mainly involving the sacroiliac joints and the axial skeleton. Other clinical findings include enthesitis, peripheral arthritis, and extra-articular organ involvements. In this regard, neurologic complications may occur in patients with AS secondary to fractures of a fused spine. Patients have risk for atlantoaxial subluxation that may lead to cervical myelopathy. Cauda equina syndrome (CES) may also be seen in severe long-standing AS [1, 2].

There are three major clinically and genetically different forms of neurofibromatosis, neurofibromatosis types 1 and 2 (NF1 and NF2), and schwannomatosis. NF1, also known as von Recklinghausen disease, is the most common type. It is a multisystem genetic disease frequently associated with neurologic, cutaneous, and orthopedic manifestations. The typical clinical manifestations of NF1 are neurofibromas, café-au-lait macules, axillary and/or inguinal freckling, and iris hamartomas [3].

## 2. Case Report

**Figure 1:**
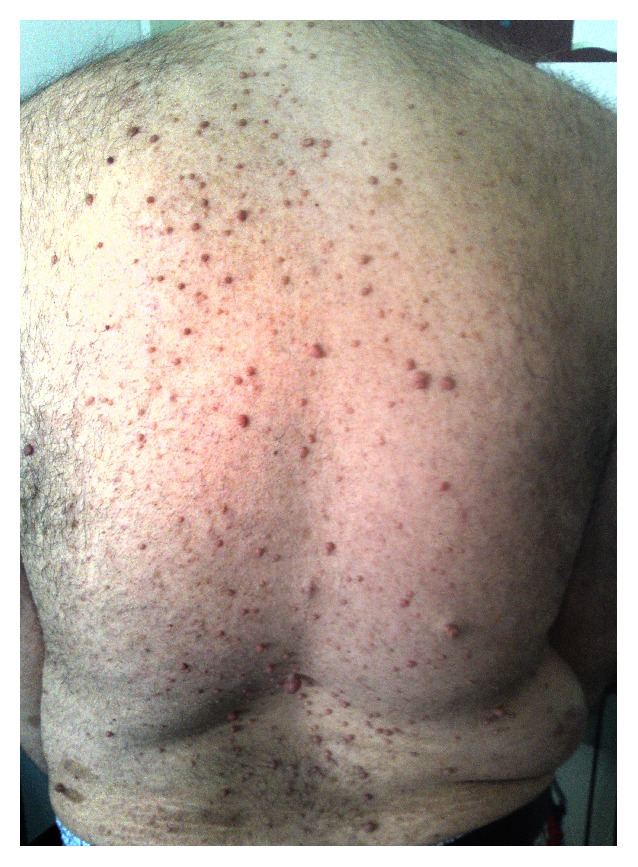
Abundant neurofibromas on dorsal side.

A 43 year-old male was admitted to the rheumatology clinic with the complaints of long-standing neck and low back pain and left lower extremity paresthesia. Neck pain was neuropathic character while low back pain was fulfilling the criteria for inflammatory back pain. Inflammatory back pain is typically characterized by following features: age of onset <40 years, insidious onset, improvement with exercise (no improvement with rest), and pain at the second half of night [4]. His past medical history included NF1 (Figure 1). Sensorimotor polyneuropathy (SM-PNP) accompanied by mixed type axonal degeneration and demyelination in the lower extremities had been detected in electromyographic examination (EMG). Then, metabolic and paraneoplastic causes of PNP had been ruled out. SM-PNP was partially controlled by gabapentin treatment.

**Figure 2:**
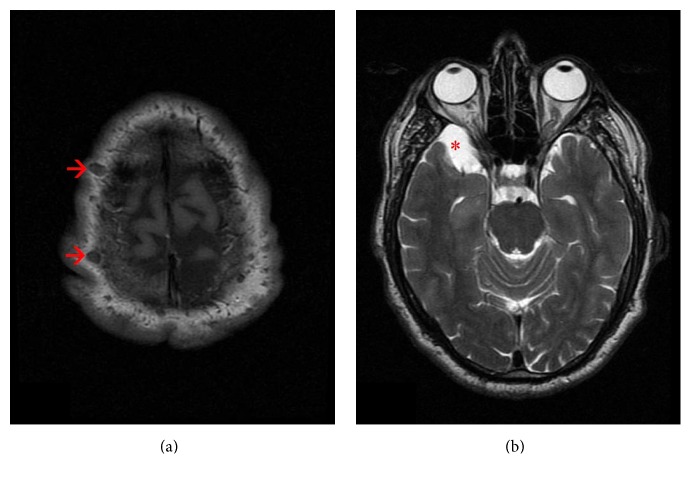
Cranial MRI findings. (a) Axial T1-weighted image depicts multiple neurofibromas on the scalp (marked by arrows). (b) Axial T2-weighted image demonstrates an arachnoid cyst in the right anterior temporal lobe (marked by an asterisk).

Because of the prior diagnosis of NF1, brain magnetic resonance imaging (MRI) had been taken. Neurofibromas on the scalp (Figure 2(a)) and 30 × 14 mm in size arachnoid cyst in the right anterior temporal lobe (Figure 2(b)) had been detected on MRI. In addition, extruded disc herniation and myelopathy due to cord compression at C4-C5 level, syringohydromyelia cavity at T12-L1 level, and biforaminal disc protrusion at L3-L4 level had been identified by MRI. Partial relief in painful SM-PNP was provided through 6-day methylprednisolone treatment at dose of 80 mg/day with gabapentin 600 mg twice a day.

**Figure 3:**
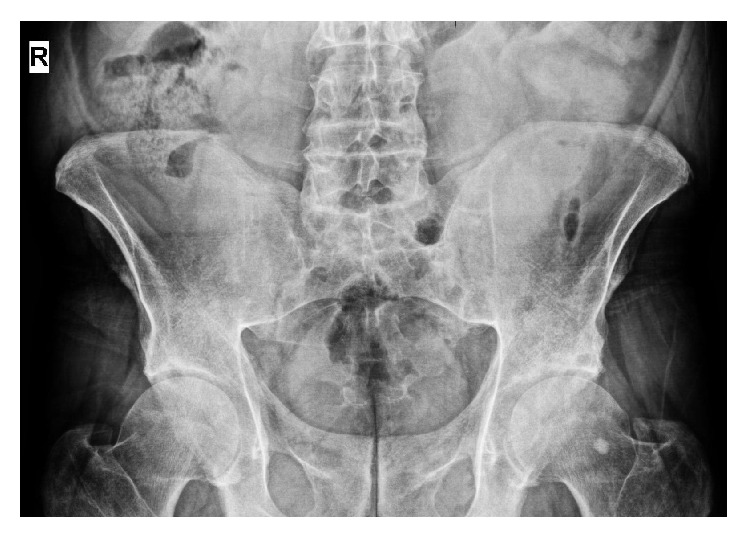
Pelvic X-ray showing bilateral grade III-IV sacroiliitis.

On physical examination, lumbar spinal motion in sagittal and frontal planes was limited. Sacroiliac joint provocation tests were positive. Neurologically, the patient exhibited mild unsteadiness during the performance of tandem gait and Rhomberg's test. Upper and lower extremity motor strengths and deep tendon reflexes appeared to be decreased slightly. On admission, hemogram, urinalysis, erythrocyte sedimentation rate, and C-reactive protein tests were within normal limits. Initial investigations also revealed normal renal and liver function. However, human leukocyte antigen B27 test was positive. Radiograph of sacroiliac joints depicted bilateral grade III-IV sacroiliitis (Figure 3). Therefore, the patient was diagnosed as AS. Meloxicam at the dose of 15 mg/day was added to patient's current treatment and the patient's symptoms abated. The patient who had stable vital signs was discharged after 3 days.

## 3. Discussion

Neurological complications in AS occur rarely and are quite variable extending from minimal joint instability of the spine to more severe and prominent clinical manifestations such as CES and cervical myelopathy [5, 6]. In the patient presented here, AS coexisted with NF1 which is characterized by the clinical triad of cutaneous findings, skeletal dysplastic deformities, and learning disabilities. NF1 has been reported in association with many infectious and chronic systemic diseases except AS [7–10].

If we take into account the epidemiological and etiopathogenetic features of both diseases, NF1 is an autosomal-dominant disease that occurs in 1 out of 3,000 births. It is induced by the inactivation of NF1 gene. NF1 gene is a tumor suppressor gene that encodes for the neurofibromin protein, a member of the Ras family. This inactivation may be a familial condition with an autosomal-dominant inheritance pattern; otherwise, it may be sporadic [10]. On the other hand, AS particularly occurs in young adults, with a peak age of onset between 20 and 30 years. Although the etiology of AS is not completely understood, it focuses on some environmental factors with a strong genetic predisposition that trigger the disease. A direct relationship between AS and the HLA-B27 gene has been determined [1]. Another possible mechanism of AS etiology is the presentation of an arthritogenic peptide from enteric bacteria via specific HLA molecules [11].

Another important point in this context is the presence of dural ectasia (DE). DE is expansion of the dural sac surrounding the spinal cord and it should also be considered as a common clinical manifestation of both diseases [6, 12] albeit not detected in our case by the present methods. DE most frequently occurs in the lumbosacral spine. It may lead to lower back pain, or neurologic deficits manifested with bowel, or bladder dysfunction. Thus, this finding is important in the differential diagnosis of both diseases.

As a result, even though the etiopathogenesis and clinical findings of AS and NF1 differ greatly, these two disorders can coexist incidentally. Upon reviewing the literature, we have noticed that this is the first case report in terms of the association of both diseases.
